# Modelling self-assessed vulnerability to HIV and its associated factors in a HIV-burdened country

**DOI:** 10.1080/17290376.2017.1387598

**Published:** 2017-10-19

**Authors:** A. F. Fagbamigbe, A. M. Lawal, E. S. Idemudia

**Affiliations:** ^a^ MSc Medical Statistics, MPDI, PhD Biostatistics is affiliated to Faculty of Human and Social Sciences, North West University, Mahikeng, South Africa; ^b^ Department of Epidemiology and Medical Statistics, Faculty of Public Health, College of Medicine, University of Ibadan, Ibadan, Nigeria; ^c^ MSc Psycology PhD Psycology is affiliated to Faculty of Human and Social Sciences, North West University, Mahikeng, South Africa; ^d^ Department of Psychology, Federal University, Oye-Ekiti, Nigeria

**Keywords:** HIV knowledge, HIV testing, risk perception, sexual risk behaviour, socio-demographics, Nigeria, Connaissance du VIH, dépistage du VIH, perception du risque, comportement sexuel à risque, socio-démographie, Nigeria

## Abstract

*Background:* Globally, individuals’ self-assessment of vulnerability to HIV infection is important to maintain safer sexual behaviour and reduce risky behaviours. However, determinants of self-perceived risk of HIV infection are not well documented and differ. We assessed the level of self-perceived vulnerability to HIV infection in Nigeria and also identified its risk factors. *Methods:* We explored a recent nationally representative data with self-reported vulnerability (‘high’, ‘low’ and ‘no risk at all’) to HIV infection as the outcome of interest. Data were weighted and association between the outcomes and the risk factors determined. We used simple ordered logit regression to model relationship between the outcome variable and risk factors, and controlled for the significant variables in multiple ordered logistic regression at 5% significance level. *Results:* About 74% had good knowledge of HIV transmission and 6% had experienced STI recently. The likelihood of assessing oneself as having ‘no risk at all’ was 50% and for ‘high chances’ was 1.6%. Self-perceived high risk of HIV was higher among those who recently experienced STI (5.6%) than those who did not (1.7%), and also higher among those who recently engaged in transactional sex and had multiple sexual partners. The odds of good knowledge of HIV transmission on high self-perceived vulnerability to HIV was 19% higher than poor knowledge (OR = 1.19, 95% CI: 1.12–1.27). Also, respondents who recently had multiple sexual partners were 72% (OR = 1.72, 95% CI: 1.60–1.86) more likely to report self as having high risk. Younger respondents aged 14–19 years had higher odds of 41% (OR = 1.41, 95% CI: 1.29–1.55) to perceive self as having high vulnerability to HIV than older respondents. *Conclusion:* High vulnerability to HIV infection was reported among younger respondents, those with history of STIS and those who engage in multiple sexual relations. Despite high level of risky sexual behaviour and good knowledge of HIV transmission and prevention found in this study, self-perceived vulnerability to HIV generally is low. For the low perception found in this study to translate to low chance of HIV infection, there is need for all stakeholders to embark on risk reduction initiatives through sexual education that would minimise risky sexual practices and ensuring availability and affordability of HIV prevention methods.

## Résumé

*Contexte:* Globalement, l'auto-évaluation des individus de la vulnérabilité à l'infection par le VIH est importante pour maintenir un comportement sexuel plus sûr et réduire les comportements à risque. Cependant, les déterminants du risque perçu d'infection à VIH ne sont pas bien documentés et diffèrent. Nous avons évalué le niveau de perception de la vulnérabilité à l'infection par le VIH au Nigéria et avons également identifié ses facteurs de risque. *Méthodes:* Nous avons exploré récemment les données représentatives au niveau national avec une vulnérabilité auto-déclarée (‘Haut’, ‘bas’ et ‘aucun risque’) à l'infection par le VIH comme résultat d'intérêt. Les données ont été pondérées et l'association entre les résultats et les facteurs de risque déterminés. Nous avons utilisé la régression logit ordonnée simple (OLR) pour modéliser la relation entre la variable de résultat et les facteurs de risque; Et contrôlé pour les variables significatives dans les OLR multiples au niveau de signification de 5%. *Résultats:* Environ 74% avaient une bonne connaissance de la transmission du VIH et 6% avaient récemment subi une IST. La probabilité de se considérer comme n'ayant aucun «risque» était de 50% et pour les «chances élevées» était de 1.6%. L'autoévaluation du risque élevé de VIH était plus élevée chez ceux qui ont récemment subi une IST (5.6%) que chez ceux qui n'en avaient pas (1,7%), et également chez ceux qui ont récemment eu des relations sexuelles transactionnelles et qui avaient de multiples partenaires sexuels. Les probabilités d'une bonne connaissance de la transmission du VIH sur une vulnérabilité élevée au VIH étaient supérieures de 19% à celles des connaissances médiocres (OR = 1.19, IC 95%: 1.12–1.27). De plus, les répondants qui ont eu récemment des partenairessexuels multiples étaient 72% (OR = 1.72, IC 95%: 1.60–1.86) plus susceptibles de se déclarer à risque élevé. Les répondants plus jeunes âgés de 14 à 19 ans étaient 41% (OR = 1.41, IC 95%: 1.29–1.55) de se percevoir comme ayant une forte vulnérabilité au VIH que les répondants âgés. Conclusion: Une forte vulnérabilité à l'infection par le VIH a été signalée chez les jeunes répondants, ceux ayant des antécédents de TSST et ceux qui se livrent à de multiples relations sexuelles. Malgré un haut niveau de comportement sexuel à risque et une bonne connaissance de la transmission et de la prévention du VIH trouvés dans cette étude, la vulnérabilité auto-perçue au VIH est généralement faible. Pour ce qui est de la faible perception que l'on trouve dans cette étude de se traduire par de faibles chances d'infection par le VIH, il faut que toutes les parties prenantes s'engagent dans des initiatives de réduction des risques grâce à une éducation sexuelle qui minimiserait les pratiques sexuelles à risque et assurant la disponibilité et l'accessibilité des méthodes de prévention du VIH.

## Introduction

Human Immunodeficiency Virus (HIV) and Acquired Immunodeficiency Syndrome (AIDS) remain clinical and public health as well as social concerns in most developing counties (Fagbamigbe, Akinyemi, Adedokun, & Bamgboye, 2011; Oladepo & Fayemi, 2011; United Nations, 2014). Globally, in 2015, estimates of 36.7 million of people were living with HIV/AIDS; 17 million accessed antiretroviral therapy and 2.1 million people turned out to be newly infected with HIV (UNAIDS, 2016). In same year in Western and Central Africa, estimates of 6.5 million of people were living with HIV/AIDS, 410, 000 new infection was recorded and 1.8 million accessed antiretroviral treatments (UNAIDS, 2016). Specifically in 2015, record has shown that in Nigeria, 3.5 million people were living with HIV/AIDS and 250,000 new HIV infection was recorded (UNAIDS, 2016) while HIV prevalence was 3.4% in 2013 (Federal Ministry of Health Nigeria, 2013). These statistics reveal how serious and endemic the issue of HIV/AIDS has remained globally and particularly in Nigeria.

The burden of HIV continues to be an issue of concern because HIV prevalence has not been significantly reduced in spite of a 35% achievement in reduction of new infections between 2005 and 2013 (UNAIDS, 2016). Unprotected heterosexual sexual activities have been reported to have accounted for about 80% of new HIV infection in Nigeria (FMoH, 2010). This suggests that many people still engage in various forms of sexual risk behaviours that could predispose them to contacting HIV infection; thus, research on perception of vulnerability to HIV infection is important until lasting solutions are found to prevalence of HIV/AIDS in Nigeria.

Perception of risk or vulnerability to health-related issues has long been noted in many theories of health protective behaviour as vital for precautionary decisions and for behaviour change (Prochaska, DiClemente, & Norcross, 1992; Rosenstock, Strecher, & Becker, 1994). For instance, in health belief model, perceived susceptibility, risk, severity, benefits and barriers were identified as predictors of safer sexual behaviour (Rosenstock, 1974). More importantly, the degree of risk perception in an individual may help him or her in adopting preventive measures in sexual behaviour. Self-perceived vulnerability to HIV infection is an individual’s belief that he or she is likely to be exposed to HIV infection.

Studies on perception of vulnerability to HIV are inadequate and not well understood in Nigeria. The few available studies have used diverse approaches in explaining the construct and reported associated factors among different categories of people. For example, Eluwa, Adebajo, Luchters, and Ahonsi (2015) investigated the prevalence and associates of HIV risk perception among homosexual persons. Also, Dibua (2009) investigated the socio-economic and socio-cultural factors predisposing individuals to HIV/AIDS in various high risk groups such as long-distance truck driver, students, commercial sex workers, single parents and street children. Mgbere et al. (2013) examined some socio-demographic and selected behavioural features as correlates of self-perception and epidemiologic ideas of risk to sexually transmitted infections and HIV infection in a population of female military personnel. Fayomi (2012) had earlier used both primary and secondary data to establish a positive relationship between poverty level and vulnerability to HIV infection. In their own study, Awosan, Ibrahim, Arisegi, and Erhiano (2014) investigated risk perception to HIV/AIDS along with knowledge, sexual life style and condom use among drivers.

Sexual practices and risk perception for HIV among youth attending National service programme have been described earlier (Amu, 2014). Lammers, van Wijnbergen, and Wilebrands (2013) explored HIV knowledge, risk perception and condom use; and made comparisons across both males and females in Nigeria. A more recent study examined the effects of Rational Emotive Health Education Programme on HIV risk perception in a sample of in-school adolescents (Onyechi, Eseadi, Okere, & Otu, 2016). However, the outcomes of these studies are conflicting due to different approaches as well as age and geographical restrictions in the sources of data used. While Awosan et al. (2014), Eluwa et al. (2015) and Lammers et al. (2013) reported poor HIV risk perception, Amu (2014) reported a moderate level of HIV risk perception in Nigeria. Therefore, a more comprehensive study is needed in order to further explain level of HIV risk perception and establish factors associating with HIV risk perception in Nigeria.

Although many factors contribute to how individuals assess themselves of risk of HIV infection, importance of HIV transmission and prevention cannot be overemphasized. In Nigeria, many studies have confirmed the relevance of HIV knowledge of prevention and transmission and as one of the contributing factors to judging a person’s level of risk to the infection (Awosan et al., 2014; Eluwa et al., 2015; Lammers et al., 2013; Osonwa et al., 2013). This suggests that HIV knowledge is crucial to the development of successful protective measures. No doubt, awareness about HIV/AIDS in Nigeria has been estimated to be 91%, which is generally high (Federal Ministry of Health Nigeria, 2013). However, it becomes worrisome that in spite of high HIV knowledge in Nigeria, the prevalence of HIV/AIDS in Nigeria was 3.4% (Federal Ministry of Health Nigeria, 2013), translating to 5.4 million infections in 2012 alone. This scenario is a pointer to the fact that other factors other than HIV knowledge are potential risk factor for HIV infection. Understanding these factors may help guide towards behaviour change in Nigeria.

In addition to HIV knowledge, the extent of engagement in sexual risk behaviours may determine how a person views his or her vulnerability to HIV infection. In Nigeria, some of the identified sexual risk behaviour associated with HIV risk perception include recent experience of symptom of STIs, multiple sexual partners, early age at first sex, being sexually active, purchase or transactional sex, receptive anal partners, multiple sexual partners, low condom use or unprotected sex and casual sex (Amu, 2014; Awosan et al., 2014; Eluwa et al., 2015; Makwe & Ahmand, 2013; Mukoro, Ogbuku, & Tabowei, 2013; Wusu, 2011). These sexual risk behaviours have been reported to have relative influences on perception of vulnerability to HIV infection among different categories of people. Nevertheless, understanding one’s vulnerability to HIV infection is incomplete if one is not sure of his or her HIV status. This can only be done through periodical HIV testing. Hence, inclusion of HIV testing as a possible associating factor in risk perception, perhaps, within the last 12 months is relevant in this study.

HIV counselling and testing is one of the preventive means severally reported to be effective in tackling the likely psychological and social features of HIV/AIDS (Federal Ministry of Health Nigeria, 2013). Being the main reason reported by respondents, HIV testing enables an individual to know his or her HIV status which can have an influence on the person’s view of his/her vulnerability to the infection. Recently, Clifton et al. (2016) reported that most black Africans and men who have sex with men; who view themselves to be at risk of HIV infection are those who have not recently been tested. Fagbamigbe et al. (2011) had earlier noted that self-assessment of risk of HIV is very important in Nigeria considering the limited availability of HIV testing. This suggests a link between knowing one’s HIV status and self-assessment of risk. Though, HIV testing is most reliable in knowing one’s status, understanding perception of vulnerability to HIV infection will be helpful in making a decision whether to go for testing or not. For instance, a Nigerian study reported that not being tested at all for HIV affects how individuals assess their risk of HIV infection (Eluwa et al., 2015). Apart from HIV knowledge and engagement in sexual risk behaviour, socio-demographics of individuals are also important in understanding their risk perception to HIV infection.

In different groups of people, many previous studies carried out in Nigeria have reported some socio-demographics such as gender, age, education, residence (Fagbamigbe et al., 2011), level of education, gender, early age at first sex in Laos PDR (Sychareun, Thomsen, Chaleunvong, & Faxelid, 2013), not exposed to peer education, being older than 25 years in Nigeria (Eluwa et al., 2015), education attainment, use of alcohol and marijuana among military personnel in Nigeria (Essien et al., 2007) as associated factors with self-perceived vulnerability to HIV/STIs. Similarly, studies previously conducted outside Nigeria confirmed some socio-demographics as relating to HIV risk perception. For example, Bastien (2008) reported that individuals with highest HIV risk perception were younger males and from rural areas. In another study, Nuri, Kerbo, and Kuti (2015) age at first sex and age group as significant correlates of HIV risk perception. The present study trails same line by examining age, sex, marital status, location of residence, wealth status, geopolitical zones, education and religion as possible socio-demographic factors determining perceived vulnerability to HIV infection.

More importantly, perceived vulnerability to HIV infection has been understudied in Nigeria. Nigeria is a country with second largest-number of people living with HIV (Central Intelligence Agency, 2014), there is always the need to understand the intricacies, associations of some socio-demographic factors, knowledge of HIV transmission and prevention, and risky sexual behaviours with perception of vulnerability to HIV infection, as this is important to curtail the scourge of HIV. Our aim is to understand the issues, determinants, levels as well as factors associated with the perception of individuals’ vulnerability to HIV infection in Nigeria. In view of this, we examined self-perception of risk to HIV infection as an outcome variable to be associated with knowledge of HIV transmission and prevention, recent contact of STIs, having multiple sexual partners, having transactional sex, current use of modern contraceptives, HIV testing and some socio-demographic factors such as age, sex, marital status, location of residence, wealth status, geopolitical zones, education and religion. The outcome of this study will offer recommendations that will assist HIV/AIDS programmers, policy-makers as well as other stakeholders in re-focusing programme so as to curtail the transmission of HIV infection in Nigeria and other developing countries.

## Materials and methods

This study used the data from Nigeria’s most recent National HIV and AIDS Reproductive Health Survey conducted in 2012 by Nigeria Federal Ministry of Health (2013). The survey was nationally representative and cross-sectional in design. The survey adopted stratified four-stage cluster probability sampling method to select women and men of reproductive ages from rural and urban households spread across areas the 36 states in Nigeria and the Federal Capital Territory, Abuja.

### Sampling

The four stages are (i) selection of local governments areas (LGAs), which include both rural and urban LGAs in each state; (ii) enumeration areas known as clusters within the selected LGAs were in the second stage; (iii) selection of households from the selected clusters and (iv) selection of individuals who are the sampling units from the households. In all, 31,235 participated in the survey. Information on knowledge of HIV/AIDS, likelihood of being HIV-infected and other reproductive health issues was collected using pretested questionnaires administered by trained interviewers.

### Data

The respondents were asked ‘How would you rate your chances of being infected with HIV?’ The responses were (a) already infected, (b) high, (c) low and (d) no risk at all. Respondents who declared themselves as already living with HIV were not included in the study. Only 25,885 respondents who had valid responses to self-reported assessment of HIV infection were included in further analysis.

### Dependent variable

The outcome variable of interest in this study is the self-reported vulnerability to HIV infection. The outcome has three valid possibilities: (a) high, (b) low and (c) no risk at all.

### Independent variables

The independent variables include knowledge of HIV transmission and prevention. Knowledge of HIV transmission and prevention were assessed using 11 knowledge-based questions. For HIV transmission, the questions are ‘How can a person get the virus that causes AIDS?’. Respondents answered ‘Yes’ or ‘No’ to ‘sexual intercourse’, ‘blood transfusion’, ‘mother to unborn child’, ‘sharing toilets’, ‘sharing sharp objects like razors’, ‘sharing needles’, ‘sharing eating utensils’, ‘mosquito bites/bed bugs’, ‘witchcraft’ and ‘hugging’. Whereas for HIV prevention, we used responses to the question: ‘What can a person do to avoid getting the virus that causes AIDS?’ Respondents answered ‘Yes’ or ‘No’ to ‘staying with one faithful uninfected partner’, ‘using condoms every time’, ‘abstaining from sex’, ‘delaying the onset of sexual intercourse’, ‘avoiding sex with commercial sex workers’, ‘reducing number of sexual partners’, ‘avoiding sex with people who have many sexual partners’ and ‘avoid sharing of sharp objects like needles, razors’, ‘praying to god’, ‘going for check-ups’ and ‘using antibiotics’, ‘seek protection from a traditional healer’.

**Fig. 1.**
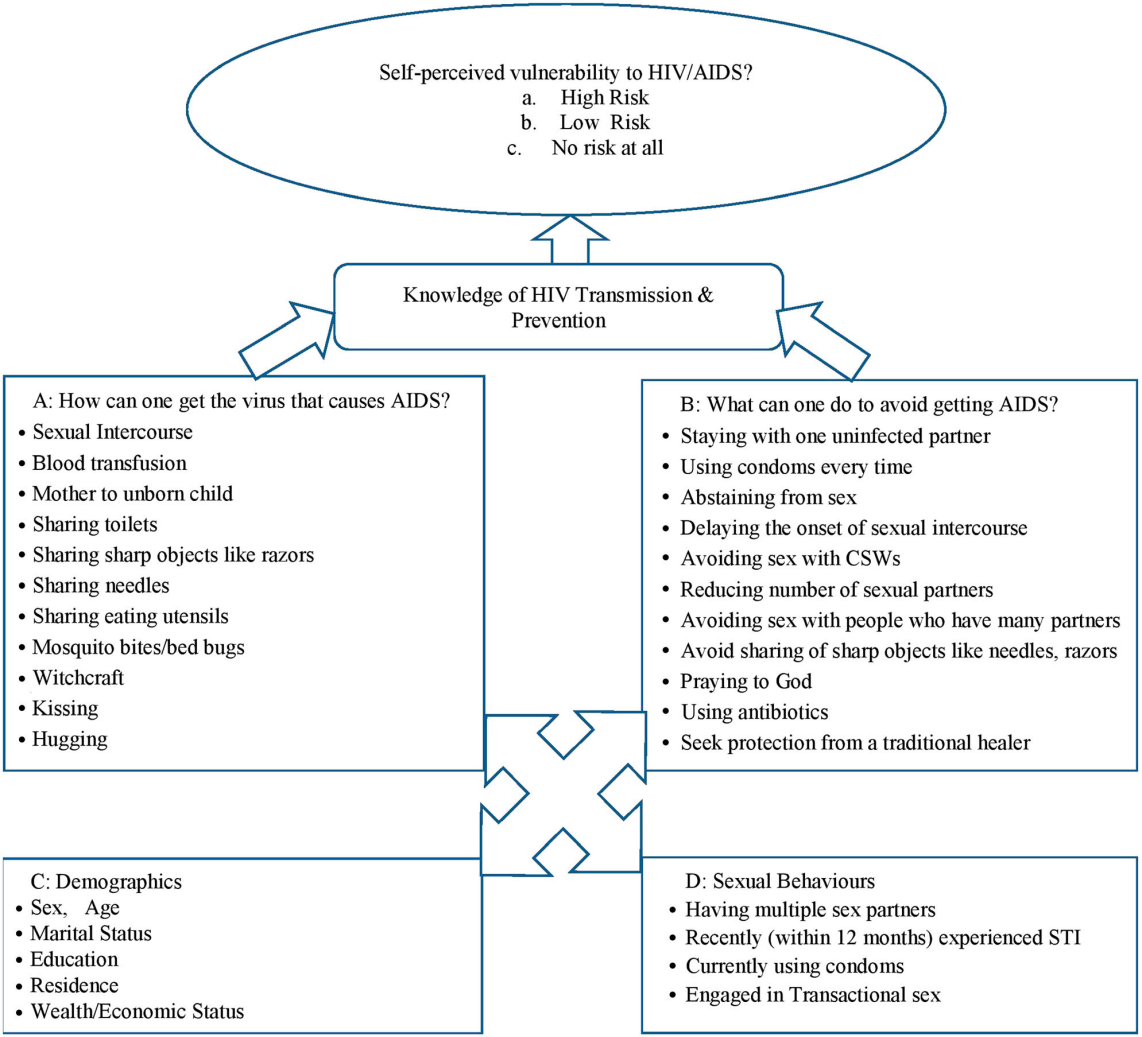
Conceptual framework.

We generated a knowledge score totalling 100%. Individuals scoring 50% or above were regarded as having good knowledge, while those with scores lower than 50% were adjudged as having poor knowledge as used earlier in an India study (George et al., 2013). The other independent variables used in this paper include ‘sex of respondents’, ‘educational attainment’, ‘economic status’, ‘location of residence’, ‘geopolitical zones’, ‘marital status’, ‘religion’ and ‘age’. Others are sexual behaviours: having multiple sex partners, recently (within 12 months) experienced STI, currently using condoms and engagement in transactional sex recently. Also, HIV testing was included as an independent variable. The conceptual framework for this study is shown in Fig. 1.

### Statistical analyses

We carried out descriptive analysis of the distribution of study participants by various explanatory variable considered and outcome of self-assessed vulnerability to HIV infection. Bivariate analyses were carried out using Pearson’s Chi-square (*χ* ^2^) test of association between the perceived vulnerability and independent variables. We used ordered logit regression to model relationship between the outcome variable and independent variables and controlled for the significant variables in multiple logit regression to determine variables associated with the study outcome. We weighted the data to reflect variations in population sizes of each state in Nigeria. We attempted to minimise the intra-cluster correlation because of the cluster nature of the data by the use of effective sample size and complex survey data analysis mechanism in Stata 12. All statistical significances were determined at 5%.

### Rationale for ordered logit regression

Ordinal logit compared with multinomial model is most ideal model in a situation when a dependent variable in a study has three or more categories and the categories can be assigned values which have a meaningful sequential order where a value is indeed ‘higher’ than the previous one. The ordered logit models are more ideal in estimating relationships between ordinal (categorical and ordered) outcome variable and explanatory variables. Rather, the multinomial logistic regression discards the ordinal information of the outcome.

The underlying score in ordered logit is that the response variable is estimated as a linear combination of the explanatory variable(s) and a set of cut off points. The likelihood of observing outcome *i* among a set of outcomes *i*, *k*, *j* is associated with the likelihood that the estimated linear combination, including a random error, is within the boundary estimated for the outcome (Long & Freese, 2006).
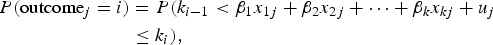
where 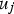 follows the assumption of logistic distribution in ordered logit. The coefficients 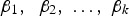 are estimated together with the cut points 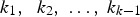
where *k* is the number of possible categories and 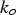 is 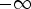 and 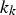 is 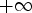.

The ordered logit regression assumes that the relationship between each pair of groups in the dependent variable groups is the same (proportional odds). Considering 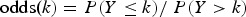 then odds 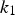 and odds 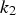 have the same ratio for all the possible combinations of the independent variables.

In ordered logit, the probability of a given observation is
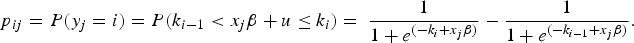
In our study, we have three possible categories: no risk at all, low risk and high risk and were coded as ‘1’, ‘2’ and ‘3’, respectively. The assumption of proportional odds was tested and we evaluated the goodness of fit of our model by comparing the likelihood values for ordered logit with multinomial models. We also predicted the chances of a respondent to assess him/herself as having ‘no risk at all’, ‘low risk’ or ‘high risk’.

### Ethical approvals

The Institutional Review Board of Nigeria National Institute of Medical Research approved the survey tools and materials. Both oral and written informed consents were obtained from all participants prior to commencement of interviews. Anonymous link test were used and respondent’s personal identifiers were not obtained. Other details have been reported earlier (Federal Ministry of Health Nigeria, 2013).

## Results

Most respondents were aged 25–49 years with 31% aged 25–34 years and 30% aged 35–49 years while 66% were either married or living with sexual partners and 63% resides in rural areas. About 74% had good knowledge of HIV transmission, 6% had experienced STI recently (within 12 months preceding the survey) while proportion who had recently engaged in transactional sex (having given or collected money or any other gift for sexual intercourse), had multiple sexual partners and were currently using modern contraceptives were 4%, 15% and 14%, respectively, as shown in first panel of Table 1. The modern methods include male and female steriliszation, pills, IUD, injectables, implants, male condom, female condom and lactational amenorrhoea method.

**Table 1.** Distribution of self-assessed vulnerability to HIV in Nigeria by respondents’ characteristics.

Characteristics	*N*	%^a^	No risk^b^ at all	Low	High	*χ*^2^ sig.
*Knowledge of HIV transmission*						
Poor	6844	26.5	57.1	41.7	1.2	0.000
Good	19,011	73.5	48.3	49.5	2.1	
*Knowledge of HIV prevention*						
Poor	5722	22.1	56.2	42.0	1.8	0.000
Good	20,133	77.9	49.1	49.0	1.9	
*Recently had STI*						
No	24,296	94.0	51.2	47.2	1.7	0.000
Yes	1559	6.0	42.8	51.6	5.6	
*Recently had multiple sex partner*						
No	22,052	85.3	53.0	45.5	1.5	0.000
Yes	3803	14.7	37.2	58.7	4.1	
*Recently had transactional sex*						
No	24,770	95.8	51.1	47.2	1.8	0.000
Yes	1085	4.2	40.9	54.2	4.9	
*Currently using MC*						
No	22,257	86.1	52.2	46.1	1.8	0.000
Yes	3598	13.9	41.4	55.9	2.7	
*Tested for HIV*						
<12	2843	11.0	48.2	49.2	2.7	0.000
Over 12	4378	16.9	44.1	53.9	2.0	
Never	18,634	72.1	52.5	45.8	1.8	
*Age*						
15–19	3907	15.1	58.3	40.1	1.6	0.000
20–24	3902	15.1	48.8	48.7	2.6	
25–34	8103	31.3	47.8	50.2	2.1	
35–49	7728	29.9	50.4	48.0	1.7	
50–64	2215	8.6	52.0	46.5	1.5	
*Sex*						
Male	13,592	52.6	50.1	48.0	2.0	0.274
Female	12,263	47.4	51.3	46.9	1.9	
*Marital status*						
Currently married	17,029	66.1	50.0	48.4	1.6	0.000
Formerly married	1013	3.9	50.8	46.2	3.0	
Never married	7712	30.0	52.1	45.5	2.4	
*Residence*						
Urban	9611	37.2	51.2	47.0	1.9	0.239
Rural	16,244	62.8	50.4	47.7	1.9	
*Wealth status*						
Poorer	10,727	41.5	52.5	45.5	2.0	0.001
Middle	4992	19.3	47.7	50.4	1.9	
Richer	10,106	39.1	50.2	48.0	1.8	
*Zone*						
North Central	6372	24.6	46.0	50.8	3.1	0.000
North East	3398	13.1	50.4	47.3	2.4	
North West	5536	21.4	61.0	37.9	1.1	
South East	1957	7.6	48.4	50.3	1.3	
South South	2680	10.4	44.5	54.1	1.3	
South West	5913	22.9	49.6	48.9	1.5	
*Education*						
No formal education	5683	22.0	54.1	44.0	1.9	0.000
Qur’anic only	6344	24.6	50.7	47.8	1.5	
Secondary	10,133	39.2	49.4	48.6	2.0	
Higher	3665	14.2	48.7	49.0	2.3	
*Religion*						
Islam	12,177	47.1	54.1	44.5	1.4	0.000
Christian	10,435	40.4	47.3	50.5	2.2	
Catholic	2827	10.9	48.6	48.5	2.9	
Other	417	1.6	48.5	49.3	2.2	
Total	25,885	100	50.7	47.5	1.9	

Note: MC: modern contraceptive.

^a^% distribution of respondents characteristics (column %).

^b^% distribution of study outcomes within each category of characteristics (row).

In all, 50.7% perceived no risk of HIV infection while 1.9% believed they had high risk of been infected. Self-reported high vulnerability to HIV infection was higher among persons with good knowledge of HIV transmission (2.1%) than those with poor knowledge (1.2%). Also, high vulnerability was higher among those who recently experienced STI (5.6%) than those who did not (1.7%), and among those who recently engaged in transactional sex and having had multiple sexual partners at 4.9% and 4.1% compared with 1.8% and 1.5%, respectively, among those who did not. The self-reported chances of HIV infection were also higher among those who got tested for HIV recently, those who were formerly married and those from North Central Nigeria. All characteristics considered were significantly related to the self-assessed vulnerability to HIV infection at 5% Chi-square test except respondent sex and rural-urban residence (second panel of Table 1).

In the bivariate analysis, the odds of assessing oneself has having ‘low risk’ compared to having ‘no risk at all’ and of having ‘High risk’ compared ‘low risk’ was higher among respondents with good knowledge of HIV transmission (OR = 1.33, 95% CI: 1.26–1.41) and good knowledge of HIV prevention (OR = 1.33, 95% CI: 1.25–1.41), recently had STI (OR = 1.47, 95% CI: 1.33–1.63), recently had multiple sexual partners (OR = 2.03, 95% CI: 1.90–2.18) and having had transactional sex recently (OR = 1.84, 95% CI: 1.64–2.05).

The odds of having higher perception of being HIV-infected was 11% times higher among currently married respondents than among the never married respondents (OR = 1.11, 95% CI: 1.05–1.17). Also, respondents from poorer households rated themselves to be more vulnerable to HIV infection (OR = 1.14, 95% CI: 1.07–1.22) compared with those from households in richer wealth quintiles. Compared with respondents from South West, those from South South had higher odds of rating themselves as more susceptible to HIV (OR = 1.34, 95% CI: 1.24–1.46) (Table 2).

**Table 2.** Unadjusted determinants of self-assessed vulnerability to HIV in Nigeria.

Characteristics	OR	95% CI	% change in Odds	*p* value
Good knowledge of HIV transmission	1.33	1.26–1.41	33.0	.000
Good knowledge of HIV prevention	1.33	1.25–1.41	33.0	.000
Recently had STI	1.47	1.33–1.63	47.0	.000
Recently had multiple sex partner months	2.03	1.90–2.18	103.0	.000
Recently had transactional sex	1.84	1.64–2.05	84.0	.000
Currently using modern contraceptive	1.58	1.48–1.69	58.0	.000
Tested for HIV				
<12	1.20	1.11–1.29	20.0	.000
Over 12	1.35	1.27–1.44	35.0	.000
Never	Ref			
Age				
15–19	1.56	1.43–1.70	56.0	.000
20–24	1.63	1.51–1.76	63.0	.000
25–34	1.45	1.34–1.56	45.0	.000
35–64	Ref			
Sex				
Male	1.04	0.99–1.09	4.0	.138
Female	Ref			
Marital				
Currently married	1.11	1.05–1.17	11.0	.000
Formerly married	1.06	0.93–1.20	6.0	.383
Never married	Ref			
Location				
Urban	Ref			
Rural	1.05	0.99–1.10	5.0	.093
Wealth status				
Poorer	1.14	1.07–1.22	14.0	.000
Middle	1.06	1.01–1.12	6.0	.028
Richer	Ref			
Zone				
North Central	1.19	1.10–1.30	19.0	.000
North East	1.12	1.03–1.22	12.0	.009
North West	0.64	0.59–0.70	−36.0	.000
South East	1.03	0.95–1.13	3.0	.445
South South	1.34	1.24–1.46	34.0	.000
South West	Ref			
Education				
No formal education	Ref			
Qur’anic only	1.10	1.02–1.18	10.0	.015
Secondary	1.19	1.11–1.27	19.0	.000
Higher	1.24	1.14–1.36	24.0	.000
Religion				
Islam	Ref			
Christian	1.36	1.29–1.43	36.0	.000
Catholic	1.15	1.06–1.23	15.0	.000
Other	1.36	1.11–1.66	36.0	.003

Controlling for other variables constant, the odds of good knowledge of HIV transmission on perception of self-vulnerability to HIV infection was OR = 1.19, 95% CI: 1.12–1.27. This implied that a respondent with good knowledge of HIV transmission is 19.1% more likely to assess him/herself as having high vulnerability to HIV infection compared with those with poor knowledge. Also, respondents who recently had multiple sexual partners were 72% (OR = 1.72, 95% CI: 1.60–1.86) more likely to report self as having high risk than reporting low or no risk at all. Also, younger respondents aged 15–19 years (OR = 1.41, 95% CI: 1.29–1.55) and those aged 20–24 years (OR = 1.43, 95% CI: 1.1.30–1.56) were more likely to assess self as having high risk of HIV infection than older respondents aged 35–64 years as shown in Table 3. The test of proportionality of odds between levels of the study outcomes was significant for the model. The goodness of fit test carried out on the model showed that the ordered logit model used in this study fitted the data more than a multinomial model as it has a lower deviance but higher likelihood.

**Table 3.** Adjusted determinants of self-assessed vulnerability to HIV in Nigeria.

Characteristics	OR	95% CI	% change in Odds	*p* value
Good KHT	1.19	1.12–1.27	19.1	.000
Good KHP	1.13	1.06–1.21	13.2	.000
Recently had STI	1.31	1.18–1.46	31.3	.000
Recently had multiple sex partner	1.72	1.60–1.86	72.1	.000
Recently had transactional sex	1.21	1.08–1.36	21.1	.002
Currently using modern contraceptive	1.25	1.16–1.35	8.3	.000
Tested for HIV				
<12	1.03	0.94–1.12	2.6	.549
Over 12	1.19	1.11–1.27	18.5	.000
Never	Ref			
Age				
15–19	1.41	1.29–1.55	41.2	.000
20–24	1.43	1.30–1.56	42.5	.000
25–34	1.27	1.15–1.41	27	.000
35–64	Ref			
Marital status				
Currently married	1.07	0.99–1.15	6.7	.093
Formerly married	1.00	0.87–1.14	−0.4	.955
Never married	Ref			
Wealth status				
Poorer	0.99	0.92–1.06	−1.2	.745
Middle	0.86	0.8–0.92	−14.2	.000
Richer	Ref			
Zone				
North Central	1.17	1.08–1.28	17.1	.000
North East	1.12	1.01–1.23	11.5	.027
North West	0.68	0.61–0.75	−32.2	.000
South East	1.09	0.99–1.2	9.0	.075
South South	1.25	1.15–1.37	25.4	.000
South West	Ref			
Education				
No formal education	Ref			
Qur’anic only	1.01	0.93–1.09	1.0	.805
Secondary	1.04	0.95–1.13	3.7	.394
Higher	0.96	0.86–1.06	−4.4	.394
Religion				
Islam	Ref			
Christian	1.04	0.97–1.12	4.3	.236
Catholic	0.90	0.82–0.99	−10.2	.025
Other	1.13	0.92–1.4	13.5	.241

In Table 4, we present the predicted probabilities of assessing self as having no risk, low risk or high risk estimated from the multiple OLR models presented in Table 3. Among respondents with poor knowledge of HIV transmission, the probability of rating self as having no risk, low risk or high risk is 0.489, 0.494 and 0.017, respectively, and it was 0.436, 0.545 and 0.017 provided that the rest of the variables are held constant at their mean values. The likelihood of assessing oneself as having ‘no risk at all’ given that all variables considered in the model are at their mean values is 50% and 1.6% for ‘high chances’.

**Table 4.** Predicted probabilities of self-assessed vulnerability to HIV in Nigeria.

Characteristics	No risk	Low	High	Sum
*Knowledge of HIV transmission*				
Poor	0.533	0.453	0.014	1.000
Good	0.489	0.494	0.017	1.000
*Knowledge of HIV prevention*				
Poor	0.524	0.461	0.015	1.000
Good	0.493	0.490	0.017	1.000
*Recently had STI*				
No	0.504	0.480	0.016	1.000
Yes	0.436	0.543	0.021	1.000
*Recently had multiple sex partner*				
No	0.521	0.464	0.015	1.000
Yes	0.387	0.588	0.026	1.000
*Recently had transactional sex*				
No	0.502	0.482	0.016	1.000
Yes	0.454	0.526	0.020	1.000
*Currently using modern contraceptive*				
No	0.508	0.476	0.016	1.000
Yes	0.452	0.528	0.020	1.000
*Tested for HIV*				
<12	0.501	0.483	0.016	1.000
Over 12	0.465	0.516	0.019	1.000
Never	0.507	0.477	0.016	1.000
*Age*				
15–19	0.559	0.428	0.013	1.000
20–24	0.473	0.508	0.018	1.000
25–34	0.471	0.511	0.018	1.000
35–64	0.500	0.484	0.016	1.000
*Sex*				
Male	0.497	0.487	0.017	1.000
Female	0.502	0.481	0.016	1.000
*Marital*				
Currently married	0.493	0.490	0.017	1.000
Formerly married	0.511	0.474	0.016	1.000
Never married	0.510	0.475	0.016	1.000
*Residence*				
Urban	0.507	0.477	0.016	1.000
Rural	0.496	0.488	0.017	1.000
*Wealth status*				
Poorer	0.484	0.499	0.017	1.000
Middle	0.487	0.496	0.017	1.000
Richer	0.522	0.463	0.015	1.000
*Zone*				
North Central	0.468	0.513	0.019	1.000
North East	0.481	0.502	0.018	1.000
North West	0.604	0.385	0.011	1.000
South East	0.486	0.496	0.017	1.000
South South	0.451	0.529	0.020	1.000
South West	0.508	0.476	0.016	1.000
*Education*				
No formal education	0.502	0.481	0.016	1.000
Qur’anic only	0.500	0.484	0.016	1.000
Secondary	0.493	0.490	0.017	1.000
Higher	0.514	0.471	0.016	1.000
*Religion*				
Islam	0.501	0.483	0.016	1.000
Christian	0.490	0.493	0.017	1.000
Catholic	0.528	0.458	0.015	1.000
Other	0.469	0.512	0.018	1.000
Total	0.500	0.484	0.016	1.000

**Fig. 2.**
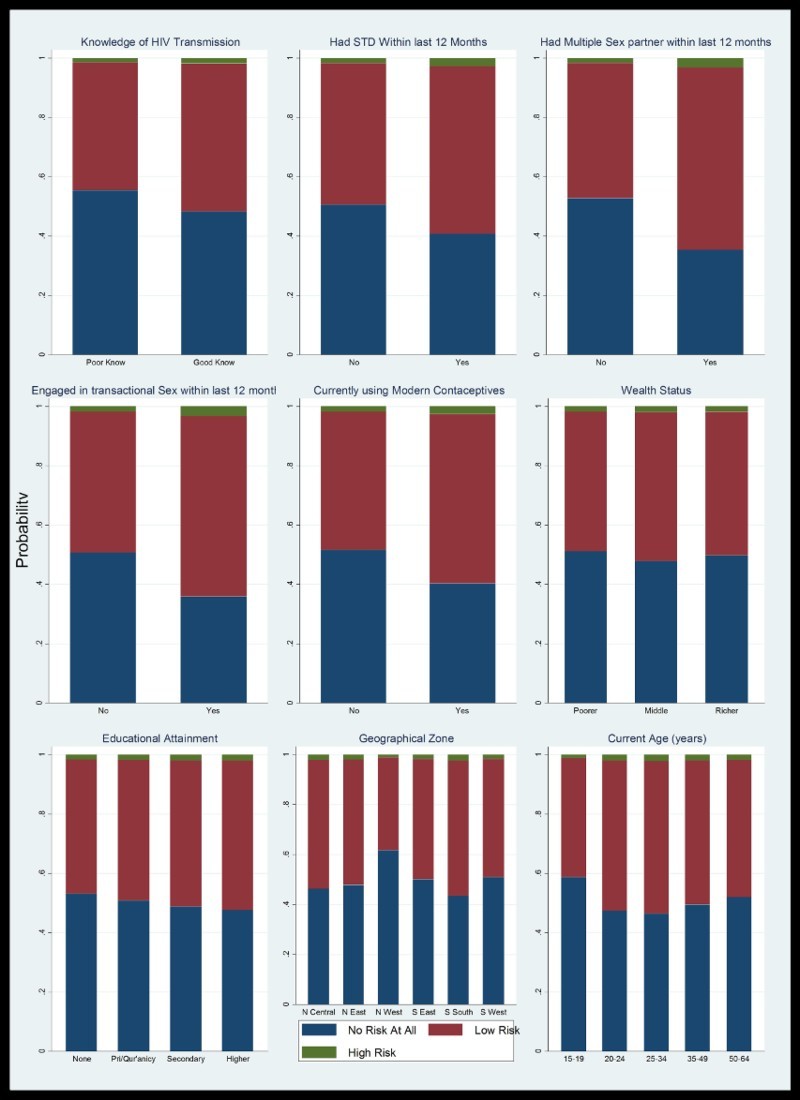
Distribution of predicted likelihoods of self-assessed vulnerabilities to HIV infection by selected characteristics in Nigeria.

The graphical distribution of the chances of assessing oneself vulnerability to HIV infection as having any of ‘no risk at all’, ‘low risk’ or ‘high risk’ is shown in Fig. 2.

## Discussion

In the study, we assessed the level of perception of vulnerability to HIV infection in Nigeria and identified knowledge of HIV transmission and prevention, HIV testing, sexual behaviours and some socio-demographics characteristics as associated factors with self-perceived vulnerability to HIV infection. Specifically, characteristics such as religion, education, geopolitical zones, wealth status, marital status, age, HIV knowledge of prevention and transmission, recent experience of STIs, having multiple sexual partners, engagement in transactional sex, current use of modern contraceptives and HIV testing were found to have significant associations with self-perception of vulnerability to HIV infection among the respondents.

### HIV knowledge and risk perception

In the current study, HIV knowledge was found to be associated with self-perceived vulnerability to HIV infection among respondents. The finding corroborates earlier reports that knowledge of HIV contributes in explaining how people assess their risk of HIV infection (Awosan et al., 2014; Eluwa et al., 2015; Lammers et al., 2013; Osonwa et al., 2013). Interestingly, we found that individuals who have good knowledge of transmission and prevention of HIV to have reported higher vulnerability than those who have poor knowledge. Our finding confirmed earlier report that although awareness of HIV/AIDS in Nigeria may be high (Federal Ministry of Health Nigeria, 2013), and despite good knowledge of HIV transmission and prevention, safer sexual practices are still very low. Thus, being more knowledgeable about HIV and AIDS is not enough for a positive behaviour change among people in Nigeria.

### Sexual risk behaviour and risk perception

Haven experienced STIs recently, had multiple sexual partners, engaged in transactional sex and current use of modern contraceptive were significantly associated with self-perceived susceptibility to HIV infection. These findings are in consonance with outcomes of previous Nigerian studies that have similarly identified these particular sexual risk behaviours as significant factors contributing to HIV risk self-perception (Amu, 2014; Awosan et al., 2014; Eluwa et al., 2015; Makwe & Ahmand, 2013; Mukoro et al., 2013; Wusu, 2011). These sexual risk behaviours appear to be foremost risk factors to HIV infection. Having untreated STI or having multiple sexual partners makes one to be at risk to HIV/STIs especially if one of the partners is infected. Also, engaging in exchange of sex for money and unprotected sex are predisposing factors for HIV infection. An individual can therefore easily judge himself to be more vulnerable to HIV, knowing fully well his degree of engagement in such risky behaviours.

### HIV testing and risk perception

In this study, we found that respondents whose last time of HIV test was over 12 months before the survey assessed themselves as more vulnerable to infection than those who did not. This finding is in agreement with prior reports that people who perceived themselves as at risk of HIV were those who have not gone for HIV test recently or never (Clifton et al., 2016; Eluwa et al., 2015). Our finding has further established the importance of HIV testing in ascertaining one’s status and preventing further risky sexual behaviour. Respondents who had long tested for HIV tend to perceive themselves as more vulnerable because of the long interval and not sure of whether they might have contracted the infection within the period. This finding therefore provides an understanding of HIV testing in the context of self-assessment of risk of HIV infection among people in Nigeria. However, it is not certain why these categories of people who perceived themselves as having high risk of HIV did not get tested. This leaves a gap for future research.

### Socio-demographics and risk perception

We found respondents age, marital status, wealth status, geopolitical zones, education and religion as significantly associated socio-demographic characteristics with self-perceived vulnerability to HIV infection. Risk perception differed on age groups with younger respondents having higher perception of HIV infection than the older respondents. This finding supports outcome of previous studies in Nigeria (Eluwa et al., 2015; Fagbamigbe et al., 2011). It is worthy of note that higher HIV risk perception found among younger respondents could be attributed to higher prevalence risky sexual behaviour among them compared to older respondents. Considering the marital status of the respondents, there was no significant difference in HIV risk perception although earlier studies have established that marital status is one of the risk factors to HIV-related consequences (Adebayo, Olukolade, Idogho, Anyanti, & Ankomah, 2013; Kposowa, 2013; Shisana, Toefy, Simbayi, Malik, & Zuma, 2004).

Wealth status was also found to have a significant association with self-perceived vulnerability to HIV infection. Specifically, respondents from households in richer wealth quintile reported higher risk than those from poorer households. The finding is in line with the study of Parkhurst (2010), which found a positive relationship between socio-economic and HIV risk perception. However, our finding is in disagreement with outcome of Madise et al. (2012) that no significant association exists between wealth status and risk of being HIV positive. A possible argument for the current finding is that individuals from richer household might have the capacity and resources to acquire more sexual partners than those from poorer household. It was also found that respondents from South South zone reported highest chances of HIV infection followed by those from North Central while respondents from North West reported lowest chances. This finding corresponds with finding of Fagbamigbe et al. (2011) that geopolitical zone is one of the significant covariates to likelihood of having HIV among men and women in Nigeria. Undoubtedly, HIV epidemic in Nigeria appears to be complex in that it is more endemic in some zones than others. This might not be disconnected from risky sexual behaviours across the geopolitical zones. This corroborates with the report that the South South zone has the highest prevalence rate of 5.5% compared to other zones (United Nations, 2014).

Respondents’ educational attainment influenced their self-perceived vulnerability to HIV infection. Highest risk perception was found among individuals with secondary education followed by those with Quranic education with least risk among respondents with higher education. The finding agrees with outcomes of earlier studies in Nigeria (Essien et al., 2007; Fagbamigbe et al., 2011; Mgbere et al., 2013). The direction of relationship between educational level and risk perception in the current study corroborates the finding of Essien et al. (2007) that an inverse relationship exists between the variables. The higher self-perceived risk found among respondents with secondary education further supported our finding that self-perceived vulnerability are higher among younger persons. This population subgroup has been reported as a critical HIV high risk group who tends not to practice safer sex (Federal Ministry of Health Nigeria, 2013; National Population Commission (Nigeria) and ICF International., 2014). We also found that religious affiliation was associated with risk perception. A major striking finding in this study is that self-perception of HIV vulnerability was higher among Christians than among Muslims. It is not certain if the prohibition of use of modern contraceptives among Catholic faithful (Fagbamigbe, Adebowale, & Morhason-Bello, 2015) and practice of polygamous among Muslims (Doctor, Findley, Afenyadu, Uzondu, & Ashir, 2013) could have influenced our finding.

However, our findings reveal no sex difference in perception of vulnerability to HIV infection. This suggests that males and females had similar tendencies in self-assessment of HIV infection vulnerability. This finding is contrary to previous report that sex difference exists in HIV risk perception (Kibombo, Neema, & Ahmed, 2007; Sychareun et al., 2013). We found that self-assessment of vulnerability to HIV infection was not associated with whether respondents reside in urban or rural area. This finding is not in line with many previous studies that have reported variation in HIV risk perception based on location of individuals (Eluwa et al., 2015; Fagbamigbe et al., 2011; Madise et al., 2012). It was expected that there would be health inequalities as well as variation in health challenges, as far as HIV is concerned, between residents of urban and rural areas.

## Conclusion

The study unravelled the intricacies in having STIs, multiple sexual partners, engaging in transactional sex and using modern contraceptive and their relationships with individuals’ self-assessment of vulnerability to HIV infection. It is particularly worrisome that persons with good knowledge of HIV perceived themselves to be more vulnerable to HIV. We conclude that knowledge of HIV transmission and prevention has not deterred engagement in risky sexual behaviour in Nigeria. This suggests that persons who assessed themselves as having low risk of HIV infection might have done otherwise had they have better HIV knowledge. Also self-perceived exposure to HIV infection was higher among younger respondents than other respondents, those with history of STDS and those who engage in multiple sexual relations.

## Recommendations

The anticipated positive behavioural change through adequate knowledge of HIV transmission and prevention is no longer sufficient to combat spread of HIV in Nigeria. There is an urgent need to encourage people, especially the young sub-population, to practice safe sexual activities. It is not unlikely that accessibility and affordability of contraceptives in Nigeria might have influenced usage of contraceptives. We recommend that HIV stakeholders and health educators should overhaul their programmes and policies to be consistent with the dynamics of HIV infection. They should develop risk reduction intervention programmes that would educate the populace on healthy sexual relationship so as to reduce the scourge of HIV/AIDS in Nigeria. Also, there is a need to ensure availability and affordability of HIV prevention methods.

## Study limitations

The secondary and cross-sectional nature of the data might have limited its scope. However, the stratified multistage cluster sampling method used in the study to select sample would have taken care of any possible sampling errors that might have arisen. Another limitation of the study is the self-report nature of data collection procedures which might have given room for exaggeration or biased responses, perhaps due to sensitivity nature of questions asked. However, multiple approaches adopted in asking similar questions in different ways might have reduced the chances of participants being biased in their responses. Generalisability of findings in this study, especially to other African countries, could be another limitation since we focused on Nigeria. Therefore, findings should be interpreted with caution and be limited to Nigerian populace.

## Contributions

AFF and AML conceived and designed the study. AFF analysed, wrote the methods and results and contributed to the introduction and discussion. AML wrote the introduction and discussion and contributed to the methods and results. ESI contributed to the introduction and discussion.
